# “Independent”—Gunn

**Published:** 1857-08

**Authors:** 


"*Independent*"—*Gunn.*—We notice a series of changes in the editorial management of "The Independent" of late. Dr. Goadby first retired, leaving the "Independent" under the editorial charge of Doctors Kane and Robinson. Dr. Kane has now retired, and Prof. Gunn, of the University of Michigan, has taken his place. In these changes we anticipate no decline in the interest of the publication, for in Prof. Gunn we think to find a worthy successor to those, who, preceding him, have assisted to make their journal one of the best in the country.

But our wonder is, at the same time, slightly excited by this last change, for it is somewhat notorious that the "Independent" has for a considerable time been engaged in a sharp controversy with the "Peninsular Journal," the latter the organ of the Faculty of the University of Michigan, among whom Prof. Gunn occupies a place. There has been not a little *sharp shooting* from both sides, and so far as we could judge, the "Independent" has never come out second best. We thought, upon first seeing Prof. G.'s name upon the cover of the "Independent," that resort had been had to the ordinary custom in *military* practice—turning captured *guns* upon the enemy from whom they were taken—and we opened the book rather expecting some *home shots*. But we were mistaken and found the gun had been christened "Peace Maker." Henceforth, we opine, the war is ended; but we do not know if hereafter we are to look upon both journals as "organs of the medical department of the Michigan University."

The "Peninsular," we see, congratulates itself upon the change, and is rather disposed, we should judge from its remarks, to set up a "rooster" as if it were conqueror in the contest. It may be that it has been able, by superior *diplomatic* talent, to accomplish what it failed to do upon the open field. Its covert fling at Dr. Goadby is hardly as manly as it might be, when we consider that its quondam enemy is no longer in a position to return the compliment.

It is hardly astonishing that they of the "Peninsular" should promise to bury the hatchet so far as the senior editor of the "Independent" is concerned, when it is remembered that he is connected with them in the Faculty of the school of which their journal is the avowed organ.

But this warfare aside; we heartily congratulate the "Independent" upon the acquisition of Prof. Gunn; we look upon it as a promise of continued and increased interest and usefulness. And if more were needed to secure success, the efforts and influence of the editorial "collaborators," among whom we are happy to see the names of a number of well known and deservedly distinguished writers and teachers in the profession, will surely be sufficient to enable the editors to attain that end. Success to you, friend "Independent," for we are confident you will deserve it. W. R. M.

